# Construction of Nanostructured Glass-Zirconia to Improve the Interface Stability of Dental Bilayer Zirconia

**DOI:** 10.3390/nano13040678

**Published:** 2023-02-09

**Authors:** Ming Zhou, Xiaoyu Zhang, Yaming Zhang, Ding Li, Zhe Zhao, Qing Wang, Kai Tang, Lina Niu, Fu Wang

**Affiliations:** 1 State Key Laboratory of Military Stomatology & National Clinical Research Center for Oral Diseases, Department of Prosthodontics, School of Stomatology, The Fourth Military Medical University, Xi’an 710032, China; 2 School of Science, Xi’an University of Posts and Telecommunications, Xi’an 710061, China

**Keywords:** zirconia, bonding force, interface stability, nanostructured glass-zirconia, dental restoration

## Abstract

Bilayer zirconia restoration is one of the most commonly used restorations in dental practice, but the high frequency of the cohesive/adhesive fracture of veneered porcelain is still a problem. This paper focuses on the development of nanostructured glass-zirconia to improve the interface stability of dental zirconia substrate and veneered porcelain. A novel SiO_2_-Li_2_O-Al_2_O_3_ (SLA) glass was prepared and infiltrated into the surface of fully sintered dental zirconia to obtain nanostructured glass-zirconia structure. The prepared glass-zirconia was analyzed with scanning electron microscopes (SEM), energy dispersive spectroscopy (EDS) and X-ray diffraction spectroscopy (XRD). The wettability, roughness and 3D morphology of zirconia were altered, and shear bonding strength (SBS) test demonstrated almost double increase in SBS values of the nanostructured glass-zirconia structure. The failure modes and microstructure characteristics also verified the improved interfacial stability. This investigation provides a promising method for enhancing the structural stability of bilayer zirconia restorations.

## 1. Introduction

With the increasing demand for aesthetics, ceramic restorations have become a preferable option in clinical practice [1]. In particular, dental zirconia ceramics are increasingly favored by patients and dentists due to their reliable mechanical performance, high wear resistance, and good biocompatibility [2,3]. Usually, the aesthetics of pure zirconia are not ideal, and a layer of porcelain is veneered on the zirconia surface to achieve better aesthetic properties. Taking advantage of the better aesthetic effect of veneering porcelain and the reliable mechanical strength of zirconia, the veneered zirconia bilayer structure has been commonly used in dental practice [4].

However, the clinical research results show that the bilayer zirconia restoration has a high probability of cohesive/adhesive fracture of veneered porcelain, which has become an important reason affecting the clinical longevity of restorations [5,6]. Many attempts have been made to enhance the interfacial stability between zirconia substrate and veneered porcelain. The efforts can be divided into three main categories: introducing an interlayer between zirconia substrate and veneered porcelain, increasing the surface roughness of zirconia, and improving the chemical activity of the zirconia [7,8,9,10]. 

Among these treatments, sandblasting is a traditional method to roughen the surface of zirconia [11], which helps to improve the mechanical embedding. However, the influence of sandblasting on bonding strength and interfacial stability is still controversial. Some studies have shown that sandblasting with appropriate parameters boosted the strength of zirconia [12], while others claimed that the phase transformation, residual stress and microcracks generated by sandblasting might have a negative impact on mechanical strength and long-term durability of zirconia restorations [13]. Ji et al. [14] used the picosecond laser to prepare a series of microtextures on the surface of zirconia to simulate the structural characteristics of natural teeth. The results showed that the hydrophilicity of zirconia with different surface textures showed a significant difference, which had a significant effect on the interfacial bond strength. The mechanical processing of zirconia surface will also provide additional mechanical interlocking [15] for the bond between zirconia and porcelain, which is conducive to the improvement of bonding force. The research on chemical etching shows that the roughness of zirconia can be improved by etching with low melting point fluoride, so as to obtain good adhesion of zirconia [16]. The above strategies modify the zirconia surface with laboratory equipment to obtain the improved surface roughness, hydrophilicity, and other properties of zirconia, providing inspirations for improving the stability of the zirconia-porcelain bilayer structure under dental laboratory conditions.

Another commonly used method is adding a liner layer between zirconia and porcelain to improve the bond strength. Nevertheless, there are different interpretations—some studies reported that the liner enhanced the bond strength, while others reported that it weakened the bond strength after long-term thermal cycling [17,18,19]. Once the restoration is cemented to the tooth, and begins to function, it is inevitably subjected to thermal, mechanical, and chemical changes. The thermal cycling resulting from the temperature changes of the oral environment induce repeated stresses at the interface of two materials. Therefore, the introduction of an interlayer increases the interface in the system of bilayer restoration, amplifies the adverse effect of the repeated interface stress with thermal cycling, and adversely affects the long-term survival rate of the restoration.

The chemically inertness of zirconia was considered to attribute to the weak bonding strength between dental zirconia and veneering porcelain [20]. Accordingly, by introducing the component with high chemical activity, the resulting zirconia surface can effectively improve the interfacial stability to the veneering porcelain. The introduction of active ingredients onto zirconia surfaces such as plasma-enhanced chemical vapor deposition (PECVD) [21], fluorapatite arrays (FA) [8], nanoscale self-limiting atomic layer deposition (ALD) of silica [7], have been demonstrated to enhance the bond strength of zirconia-porcelain significantly.

Recent literatures have reported some novel methods of surface modification of zirconia, such as fusion sputtering and glass-ceramic spray deposition, which can be easily implemented in the dental laboratory [22,23,24]. These novel surface modification techniques produce a dense coating layer of lithium disilicate glass-ceramic on the surface of zirconia. The coating forms an extensive micro-mechanical interlock with zirconia and increases its hydrophilicity, thus improving the bond strength between zirconia and resin cement.

The major structural difference between zirconia polycrystalline ceramics and glass ceramics is the absence of the glass phase, which makes zirconia chemically inert. A series of studies on graded zirconia by Zhang et al. [25,26,27,28,29] showed that the introduction of glass components into zirconia surface can significantly improve the fracture strength and adhesion to veneered porcelain. This concept has been tried on the laboratory-prepared zirconia which was presintered to reserve porous structure so that the glass could infiltrate into the surface to form a glass-zirconia composite structure. However, this has not been achieved on the commercial dental zirconia which is already fully sintered into a dense structure without any pore inside, which is essential to ensure adequate strength of the substrate.

In this study, a novel nanoscale Si-Li-Al glass was developed to infiltrate on a commercial dental zirconia to construct nanostructured glass-zirconia structure. The roughness, wettability, microstructure, and phase composition of the nanostructured glass-zirconia were analyzed. Shear bonding strength and the interface characteristics of porcelain and nanostructured glass-zirconia were evaluated.

## 2. Materials and Methods

### 2.1. Preparation of Nanoscale Glass Powder

The SiO_2_-Li_2_O-Al_2_O_3_ (SLA) glass was prepared following a melt-diffusion strategy. The starting materials and constituent ratios are shown in Table 1. The compositions were mixed with alcohol and ball-milled for 2 h to obtain a well-mixed slurry. The slurry was dried in an oven (WHL-25AB, Taisite, Tianjin, China) at 80 °C for 12 h and then heated in a platinum crucible with a high temperature electric furnace (NWTX-1600, Luoyang, China). The temperature was increased to a target temperature of 1500 °C at a rate of 10 °C·min^−1^. After holding for 2 h, the molten mixture was quenched in deionized water and then ball-milled with alcohol for 24 h to obtain the SLA glass powder with nanoscale particles. The size distribution was analyzed by the Nano Measurer 1.2 Software (Department of Chemistry, Fudan University, Shanghai, China) from the scanning electron microscopy (SEM) images.

### 2.2. Construction of Nanostructured Glass-Zirconia

The zirconia specimens were prepared from commercial dental zirconia blocks (Zenostar, Weiland dental, Pforzheim, Germany) using CAD/CAM technique and fully sintered following the manufacturer’s recommended procedure (Table 2). The sintered zirconia specimens were polished with 240, 400, 600, 800, 1200, and 2000 grits silicon carbide papers, and then cleaned by sonication in ethanol for 5 min. The SLA glass powder was mixed with deionized water (mass ratio of 1:3) to form a slurry. Each zirconia specimen was coated with a uniform layer of glass slurry, with weight of 0.05 g. Then, the specimens were heated to 1400 °C at a rate of 10 °C·min^−1^ and held for 1.5 h. Then, the nanostructured glass-zirconia (ngZir) was obtained. Some sintered zirconia specimens without any further treatments were also made as non-treat control (Zir) for comparison.

### 2.3. Characterization of Nanostructured Glass-Zirconia

#### 2.3.1. Observation of the Microscopic Morphology

The morphology and microstructure of glass powder, non-treat zirconia (Zir) and nanostructured glass-zirconia (ngZir) were observed with SEM (S-4800, Hitachi, Tokyo, Japan). The distribution of glass in ngZir specimens was detected. All specimens were ultrasonically cleaned and sputter-coated with platinum (MC 1000, Hitachi, Tokyo, Japan).

#### 2.3.2. X-ray Diffraction Spectroscopy (XRD)

Zir and ngZir specimens were analyzed with XRD (Smartlab, Japan) to examine the phase composition. The powder diffraction patterns from the International Center for Diffraction Data (ICDD) were used to compare and analyze the XRD peaks of the specimens. The scans were carried out with Cu Kα radiation from 10° to 80° with a scan rate of 10°·min^−1^. The percentage of monoclinic phase of ngZir was determined with grazing incidence X-ray diffraction (GIXRD). The angle of incidence was set at 1° to measure the glass-zirconia layer. The percentage of monoclinic phase (*V_m_*) was determined as follows [30,31]:

(1) $$V_{m}=\frac{1.311\times \left(I_{m}^{\left(-111\right)}+I_{m}^{\left(111\right)}\right)}{1.311\times \left(I_{m}^{\left(-111\right)}+I_{m}^{\left(111\right)}\right)+I_{t}^{\left(101\right)}}$$

where *I_m_* and *I_t_* are the peak intensities of monoclinic and tetragonal zirconia that could be read directly. Since the ngZir contains only tetragonal and monoclinic zirconia, the percentage of tetragonal phase (*V_t_*) can be calculated by *V_t_* = l − *V_m_*.

#### 2.3.3. Roughness Assessment

Specimens of both groups were ultrasonically cleaned with ethanol for 10 min and dried at 80 °C for 2 h before the roughness assessment. A non-contact optical profilometer (ST 400, Nanovea, Irvine, CA, USA) was used to obtain the surface morphology (step size of 10 μm, scan speed of 0.25 mm·s^−1^ and frequency of 100 Hz). The average surface roughness (Ra) and arithmetical mean height of the scale limited surface (Sa) were analyzed and calculated with professional 3D software (7.4.8737, Nanovea, Irvine, CA, USA).

#### 2.3.4. Wettability

The wettability of specimen was assessed by static contact angle evaluation using a contact angle instrument (Dataphysics DCAT21, Filderstadt, Germany). Distilled water droplets (~10 μL) were dropped onto the surface of each specimen. The image of water droplet shape was captured using an image acquisition device when the water droplet stabilizes. The image in JPEG format was analyzed with ImageJ for the determination of the contact angle. Ten specimens of each group were evaluated to obtain the average value of contact angles.

### 2.4. Fabrication of Bilayer Zirconia Specimens

Forty sintered zirconia specimens were prepared and divided into two parts. Twenty zirconia specimens were glass infiltrated with SLA glass powder to construct nanostructured glass-zirconia, and the other twenty zirconia specimens were sandblasted with 27 μm aluminum oxide particles in a fine sand-blaster (Basic classic, Renfert, Hilzingen, Germany). Two veneering techniques were used including the hand-layering method and the heat-pressing method. For the hand-layering method, a fluorapatite porcelain (Dentin A2, Ivoclar Vivadent, Schaan, Liechtenstein) was applied on the surface of specimens via the silicone mold with the attempt to keep identical contact surface and dimension of the specimens (a cube with sides of length about 4 mm). For the heat pressing method, polymethyl methacrylate (PMMA) blocks (a cube with sides of length about 4 mm) were fixed on top of the zirconia and invested using rapid investment materials (IPS Press VEST, Ivoclar Vivadent, Schaan, Liechtenstein). Consecutively, the invested molds were subjected to the burn out processes and the space created by the burn-out of the PMMA pattern was then filled by the veneering porcelain (IPS e.max ZirPress, Ivoclar Vivadent) in the specific furnace (EP 3010, Ivoclar Vivadent, Schaan, Liechtenstein) following the manufacture’s guidelines. The grouping situation is shown in Table 3. In each bilayer zirconia specimen, the area of zirconia covered by the veneered porcelain is measured to obtain an accurate bonding area.

### 2.5. Shear Bonding Strength (SBS) and Failure Mode Analysis

The specimens were tested using a universal testing machine (AG-10, Shimadzu Corporation, Kyoto, Japan) with a vertical compressive load at the junction of zirconia and porcelain. The crosshead speed was set at 0.5 mm·min^−1^. All specimens were tested and the fracture loads were recorded. The bond strength was calculated as follows:

σ = F/A(2)

where F is fracture load (N) and A refers to bonding area (mm^2^).

The failure mode between zirconia and the porcelain was inspected with stereomicroscope (Stemi 508, Zeiss, Jena, Germany) and classified as adhesive failure, cohesive failure, and mixed failure, according to the location of failure.

### 2.6. Evaluation of Bonding Interface

The bilayer zirconia-porcelain specimens were cut with a diamond saw to expose the cross-sectional surface of bonding interface between zirconia and porcelain. The cut marks were removed using sandpapers of 400, 800, 1200, 2000 grit and diamond slurry of 2.5, 1, and 0.25 μm successively. The SEM and backscattered electron (BSE) were used to observe the microscopic morphology of the interface, and energy dispersive spectroscopy (EDS) was used to perform semi-quantitative analysis of elemental distribution at the interface.

### 2.7. Statistical Analysis

In this study, the data of different groups were analyzed with SPSS 27.0 (IBM Corporation, Armonk, NY, USA), and the significance level was set at *p* < 0.05. One-way analysis of variance (ANOVA) and post hoc Tukey tests was used to investigate the statistical difference.

## 3. Results and Discussion

**Figure 1:**
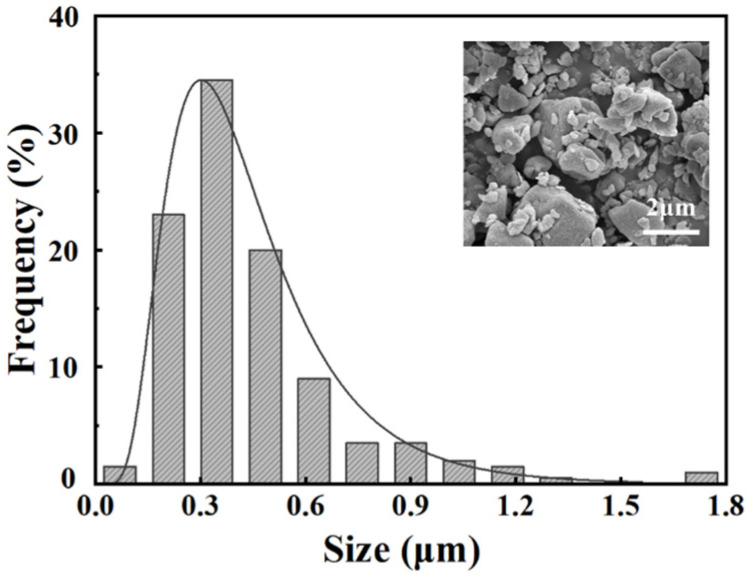
The size distribution and morphology of the nano-glass powder.

The morphology and particle size distribution of SLA glass powders are shown in Figure 1. After the designed procedure of ball-milling, the SLA glass powder displays a nanoscale size of 0.44 ± 0.26 μm and a narrow distribution range. The smaller particle size of the glass powder can increase the contact area between the glass slurry and zirconia in the process of preparing nanostructured glass-zirconia.

**Figure 2:**
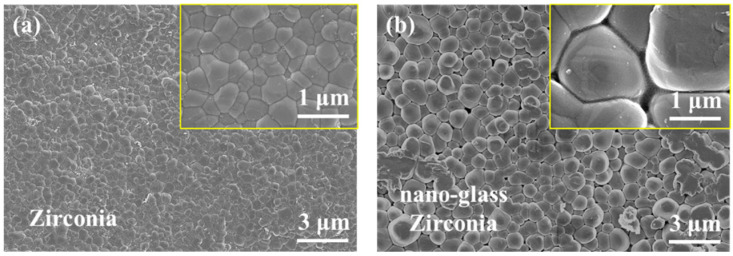
Typical SEM images of zirconia surface morphology: zirconia sintered following the recommended procedure (**a**) and sintered zirconia after modification (**b**).

According to the literature [32,33], the sintered zirconia achieves a near-complete dense state with a density of 6.0–6.1 g·cm^−3^ and an open porosity of approximately 0. Figure 2a exhibits the representative polygonal zirconia grains which are closely attached to each other. No obvious pores can be observed at the boundaries of zirconia grains. After glass infiltration, the size of zirconia grains presented increasing tendency, and nanoscale spaces were formed and occupied by glass between the zirconia grains, especially at the triple junction of the grains (Figure 2b). After micro analysis, the size of pores ranged from 84.3 to 20.4 nm.

The dense structure and stable chemical properties of zirconia make it difficult to form an effective mechanical embedding effect or chemical bond with veneered porcelain. The formation of pores makes it possible to form mechanical interlocking between porcelain and zirconia, and the introduction of nano-glass improves the chemical activity of zirconia. Therefore, the formation of nanostructured glass-zirconia is beneficial to enhancing the bonding strength between zirconia and veneered porcelain [9,34,35].

**Figure 3:**
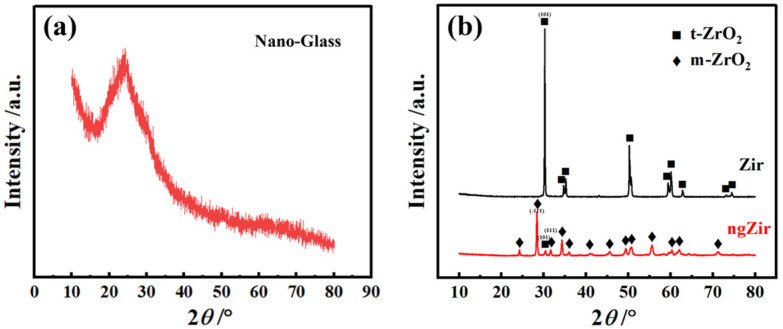
XRD patterns of the SLA glass powder (**a**) and zirconia before and after modification (**b**).

The XRD patterns of SLA glass powder, zirconia and nanostructured glass-zirconia are shown in Figure 3. With the addition of yttrium oxide, zirconia is maintained in the form of tetragonal zirconia polycrystal (*t*-phase) (Zir in Figure 3b). After modification, diffraction patterns of ngZir displayed peaks corresponding to the *t*-phase (ICDD 70-4427) and monoclinic (*m*) phase (ICDD 81-1314) zirconia. The surface layer of the ngZir specimen is composed of 90.8 vol% *m*-ZrO_2_ and 9.2 vol% *t*-ZrO_2_.

**Figure 4:**
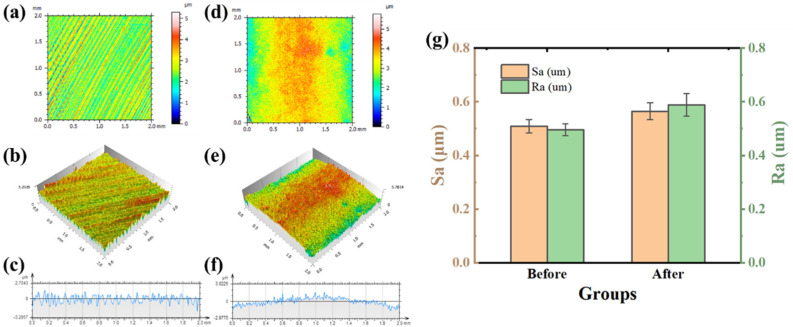
Morphology and roughness of zirconia before and after modification. 2D morphology of zirconia (**a**), 3D morphology of zirconia (**b**), section profile of zirconia (**c**), the 2D morphology, 3D morphology, and section profile of modified zirconia, respectively (**d**–**f**). The contrast of roughness before and after modification of zirconia (**g**).

Figure 4 shows the 2D and 3D morphology, section profile and roughness (Ra, Sa) of zirconia before and after modification. In Zir group, the machining marks produced during CAD/CAM processing rendered as parallel and superficial furrows which can be seen in Figure 4a. After modification, the superficial furrows disappear, and were replaced by the more prominent ridges. It can be seen that the 3D morphologies in Figure 4c,d present the same characteristics corresponding to the surface morphologies. In Figure 4e,f, the section profile gives a visual comparison of the undulating surface structure in two groups. Analysis of the surface morphology shows that group ngZir had higher Sa and Ra values than group Zir (*p* < 0.05). The results indicated that the glass infiltration in ngZir group altered the morphology and improved the roughness. It is reported that the improvement of roughness could enhance the SBS values between zirconia and porcelain [10,14,15]. Therefore, the increase of Ra and Sa values on the modified zirconia surface would be beneficial to the stability of the zirconia-porcelain structure.

**Figure 5:**
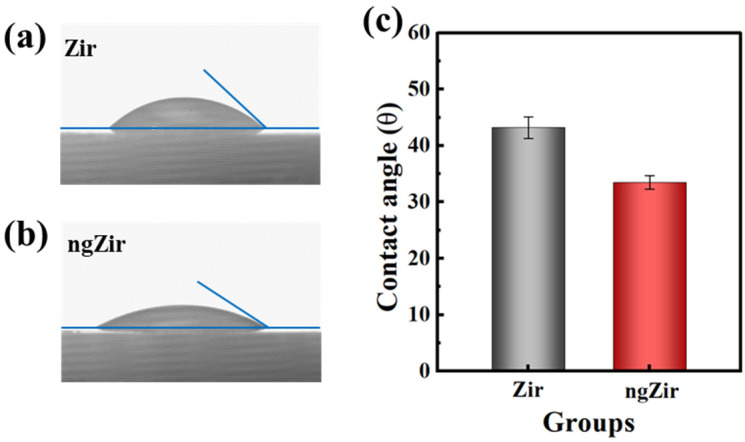
The contact angles of Zir (**a**) and ngZir (**b**), and the contrast of contact angles (**c**) before and after modification of zirconia.

During the fabrication of bilayer zirconia restorations in clinical practice, dental porcelain is veneered to zirconia at a temperature above its melting point, and the melted porcelain contacts zirconia surface in the liquid form. Therefore, the wettability of the zirconia surface is crucial to the interfacial stability of porcelain and zirconia [14,21]. Figure 5 shows the static contact angle of Zir and ngZir specimens with distilled water. The ngZir group showed higher contact angle values (43.2 ± 1.9°) than Zir group (33.5 ± 1.2°) (*p* < 0.05). Based on Young and Neumann’s equation, the contact angle of the material is inversely proportional to the surface energy [36]. As a smaller contact angle tends to need less energy and imply better wettability, construction of nanoscale glass-zirconia significantly improved the surface wettability.

**Figure 6:**
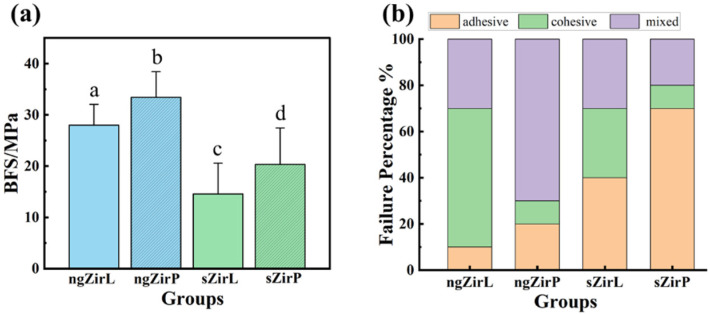
The SBS values (**a**) and failure mode (**b**) of different groups.

Figure 6 shows the results of SBS test and the fracture pattern of different groups. The ngZirP group has the highest SBS values (33.43 ± 4.99 MPa), while the sZirL group has the lowest value (14.58 ± 5.98 MPa). Compared with sandblasted zirconia (sZirL and sZirP), the bonding strength after constructing the nanostructured glass-zirconia microstructure has almost doubled. The increased SBS may be attributed to the changes in surface structure and morphology, roughness, and wettability caused by glass infiltration. Nanoscale pores are formed on the surface of ngZir and are filled with glass components, allowing for more mechanical interlocking with the veneered porcelain. In addition, ngZir has a higher roughness and better wettability, both of which contribute to a higher bond strength with the decorated porcelain. There was also a significant difference between the SBS values of different veneering methods. Compared with method of hand-layering, heat pressing veneering technique leads to almost no internal structural defects of porcelain, which also contributes to the improvement of SBS.

After the SBS test, the fracture patterns were recorded and classified according to the following criteria [7,16,37]:

(a) Adhesive failure: the failure occurred at interface of zirconia and porcelain.
(b) Cohesive failure: the failure occurred inside the porcelain.
(c) Mixed failure: both a and b failure modes exist simultaneously.

All three types of fracture patterns were observed in all groups. The proportion of adhesive failure was lower in the ngZirL and ngZirP groups, which also indicates better interface stability.

For ngZirL and sZirL groups, the same veneering methods were adopted, therefore the higher SBS values and lower occurrence of adhesive failure in ngZirL group were due to the development of nanostructured glass-zirconia structure. The same results can also be found in the ngZirP and sZirP groups, indicating that the nanostructured glass-zirconia surface significantly improved the bond strength between zirconia and porcelain. This conclusion is consistent with the results in previous literatures [38,39].

**Figure 7:**
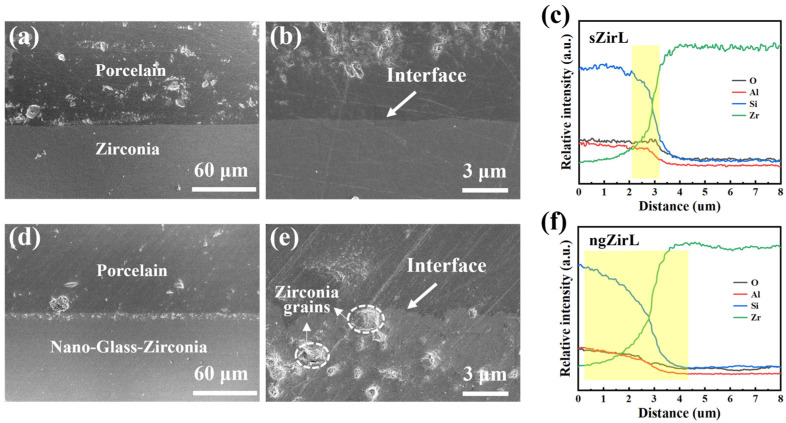
The SEM images and EDS line scan results at the interface. The SEM images of sZirL at the interface (**a**,**b**), the EDS line scan results of O, Al, Si and Zr at the interface (**c**). The SEM images of ngZirL at the interface (**d**,**e**), the EDS line scan results of O, Al, Si, and Zr at the interface (**f**).

The bonding interface between porcelain and zirconia was analyzed with SEM and EDS. In the sZirL group, the interface tended to be more of a straight line (Figure 7a,b), whereas the ngZirL group had an interlaced interface (Figure 7d,e), which is consistent with the higher roughness of the ngZir surface. Some partially exposed zirconia grains encapsulated by the glass matrix can be observed in the ngZirL group. This confirms the existence of nanostructured glass-zirconia composite structure in the subsurface layer. The results of EDS line scan analysis demonstrated that both groups formed an elemental transition region between zirconia and porcelain with continuous variation of elemental content (yellow region in Figure 7c,f). However, the ngZirL group showed a wider range of transition regions at the interface and a smoother variation in elemental content, indicating a larger crossover region at the interface, which was favorable for interface stability.

**Figure 8:**
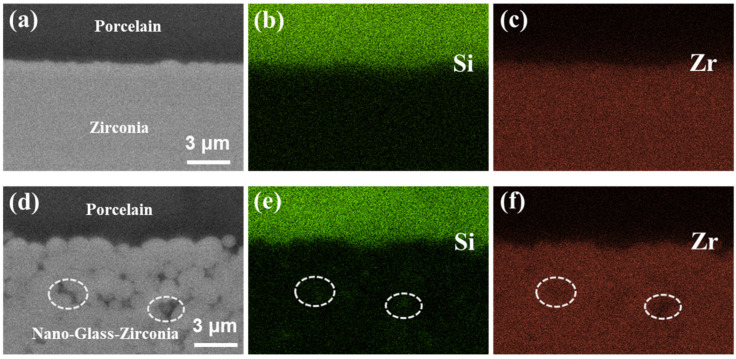
The BSE images and EDS mapping at the interface region. The BSE images of sZirL (**a**) and ngZirL (**d**). The EDS mapping of Si (**b**) and Zr (**c**) at the interface of sZirL. The EDS mapping of Si (**e**) and Zr (**f**) at the interface of ngZirL.

Figure 8 shows the BSE images at the interface of sZirL and ngZirL. In the sZirL group, the zirconia substrate exhibited homogeneous structure and composition at the region of interface, while in the ngZirL group, grain contours and darker areas (white circles) among grains could be observed, which indicated the presence of different compositions. The elemental mapping analysis of the corresponding areas displayed a homogeneous distribution of Si and Zr at the zirconia substrate in the sZirL group. In the ngZirL group, the enhancement of the Si signal and the attenuation of the Zr signal can be detected at the dark area (Figure 8e,f).

In this study, nanostructured glass-zirconia was constructed on fully sintered commercial dental zirconia by infiltrating a novel SLA glass. The nanostructured glass-zirconia has higher chemical activity due to the introduction of glass components. After modification, ngZir has higher roughness and better wettability. Due to the extensive mechanical interlocking between ngZir and veneer porcelain, a wider range of elemental transition area is formed at the interface. The above factors lead to the significant increase in the SBS value. Analysis of the fracture patterns also showed that the proportion of adhesive failures was significantly reduced. Therefore, it can be concluded from the above results that the construction of nanostructured glass-zirconia significantly improves the structural durability of zirconia bilayer restorations. In addition, the zirconia modification method used in this study can be operated simply and conveniently, and can be easily implemented in the dental laboratory.

## 4. Conclusions

In conclusion, the construction of nanostructured glass-zirconia on dental zirconia ceramics significantly improves the chemical activity, roughness and wettability of zirconia. Compared with sandblasting, nanostructured glass-zirconia broadens the elemental transition region and increases the bond strength between zirconia and porcelain by approximately double. This study provides a practical, effective and promising method for improving the interface stability of bilayer zirconia restorations.

## Figures and Tables

**Table 1:** The composition of glass powder (mol.%).

SiO_2_	Li_2_CO_3_	Al_2_O_3_	K_2_CO_3_	ZrO_2_	P_2_O_5_	Others
58.0	27.0	10.0	1.8	1.5	1.2	0.5

**Table 2:** The recommended sintering procedure.

Temperature (°C)	20~900	900~900	900~1500	1500~1500	1500~900	900~20
Time (h)	1.5	0.5	3	2	1	1.8

**Table 3:** The specific grouping situation.

Groups	Surface Treatment	Veneering Techniques
A (ngZirL)	Glass infiltration	hand-layering
B (ngZirP)	Glass infiltration	heat-pressing
C (sZirL)	Sandblast	hand-layering
D (sZirP)	Sandblast	heat-pressing

## Data Availability

The data presented in this study are available on request from the corresponding author.

## References

[1] Spitznagel F.A., Boldt J., Gierthmuehlen P.C. (2018). CAD/CAM ceramic restorative materials for natural teeth. J. Dent. Res..

[2] Zhou M., Meng M., Chai Z.Z., Zhang Y.M., Li D., Niu L.A., Jia Y.M., Zhang S.F., Wang F. (2022). Dynamic wear characteristics and fracture strength of high-translucent monolithic zirconia crowns. Ceram. Int..

[3] Shelar P., Abdolvand H., Butler S. (2021). On the behaviour of zirconia-based dental materials: A review. J. Mech. Behav. Biomed. Mater..

[4] Shahmiri R., Standard O.C., Hart J.N., Sorrell C.C. (2018). Optical properties of zirconia ceramics for esthetic dental restorations: A systematic review. J. Prosthet. Dent..

[5] De Lima E., Meira J.B.C., Özcan M., Cesar P.F. (2015). Chipping of veneering ceramics in zirconium dioxide fixed dental prosthesis. Curr. Oral Health Rep..

[6] Marefati M.T., Hadian A.M., Hooshmand T., Yekta B.E., Koohkan R. (2018). Wettability of zirconia by feldspathic veneer in dental restorations: Effect of firing atmosphere and surface roughness. Ceram. Int..

[7] Yan Y., Ji Y., Yan J., Hu X., Zhang Q., Liu M., Zhang F. (2021). Atomic layer deposition SiO_2_ films over dental ZrO_2_ towards strong adhesive to resin. J. Mech. Behav. Biomed. Mater..

[8] Li K., Kou H., Rao J., Liu C., Ning C. (2021). Fabrication of enamel-like structure on polymer-infiltrated zirconia ceramics. Dent. Mater..

[9] Silva-Herzog Rivera D., Pozos-Guillen A., Aragon-Pina A., Cerda-Cristerna B.I., Masuoka-Ito D., Sanchez-Vargas L.O. (2020). Glass coatings to enhance the interfacial bond strength between veneering ceramic and zirconia. Odontology.

[10] Abdullah A.O., Hui Y., Sun X., Pollington S., Muhammed F.K., Liu Y. (2019). Effects of different surface treatments on the shear bond strength of veneering ceramic materials to zirconia. J. Adv. Prosthodont..

[11] Moon J.E., Kim S.H., Lee J.B., Han J.S., Yeo I.S., Ha S.R. (2016). Effects of airborne-particle abrasion protocol choice on the surface characteristics of monolithic zirconia materials and the shear bond strength of resin cement. Ceram. Int..

[12] Garcia Fonseca R., de Oliveira Abi-Rached F., dos Santos Nunes Reis J.M., Rambaldi E., Baldissara P. (2013). Effect of particle size on the flexural strength and phase transformation of an airborne-particle abraded yttria-stabilized tetragonal zirconia polycrystal ceramic. J. Prosthet. Dent..

[13] Kim H.K., Yoo K.W., Kim S.J., Jung C.H. (2021). Phase transformations and subsurface changes in three dental zirconia grades after sandblasting with various Al_2_O_3_ particle sizes. Materials.

[14] Ji M., Xu J., Chen M., El Mansori M. (2020). Enhanced hydrophilicity and tribological behavior of dental zirconia ceramics based on picosecond laser surface texturing. Ceram. Int..

[15] Santos R.L.P., Silva F.S., Nascimento R.M., Souza J.C.M., Motta F.V., Carvalho O., Henriques B. (2016). Shear bond strength of veneering porcelain to zirconia: Effect of surface treatment by CNC-milling and composite layer deposition on zirconia. J. Mech. Behav. Biomed. Mater..

[16] Ruyter E.I., Vajeeston N., Knarvang T., Kvam K. (2017). A novel etching technique for surface treatment of zirconia ceramics to improve adhesion of resin-based luting cements. Acta Biomater. Odontol. Scand..

[17] Yoon H.I., Yeo I.S., Yi Y.J., Kim S.H., Lee J.B., Han J.S. (2015). Effect of various intermediate ceramic layers on the interfacial stability of zirconia core and veneering ceramics. Acta Odontol. Scand..

[18] Kim S.H., Park C.J., Cho L.R., Huh Y.H. (2018). Evaluation of the ceramic liner bonding effect between zirconia and lithium disilicate. J. Prosthet. Dent..

[19] Wang G., Zhang S., Bian C., Kong H. (2014). Effect of zirconia surface treatment on zirconia/veneer interfacial toughness evaluated by fracture mechanics method. J. Dent..

[20] Scaminaci Russo D., Cinelli F., Sarti C., Giachetti L. (2019). Adhesion to zirconia: A systematic review of current conditioning methods and bonding materials. Dent. J..

[21] Bitencourt S.B., Dos Santos D.M., da Silva E.V.F., Barao V.A.R., Rangel E.C., da Cruz N.C., de Souza G.M., Goiato M.C., Pesqueira A.A. (2018). Characterisation of a new plasma-enhanced film to improve shear bond strength between zirconia and veneering ceramic. Mat. Sci. Eng. C Mater..

[22] Peng T.Y., Kang C.M., Feng S.W., Hung C.Y., Iwaguro S., Lin D.J. (2022). Effects of glass-ceramic spray deposition manipulation on the surface characteristics of zirconia dental restorations. Ceram. Int..

[23] Ali N., Safwat A., Aboushelib M. (2019). The effect of fusion sputtering surface treatment on microshear bond strength of zirconia and MDP-containing resin cement. Dent. Mater..

[24] Aboushelib M.N. (2012). Fusion sputtering for bonding to zirconia-based materials. J. Adhes. Dent..

[25] Zhang Y., Kim J.W. (2009). Graded structures for damage resistant and aesthetic all-ceramic restorations. Dent. Mater..

[26] Zhang Y., Chai H., Lee J.J.W., Lawn B.R. (2012). Chipping resistance of graded zirconia ceramics for dental crowns. J. Dent. Res..

[27] Chai H., Lee J.J.W., Mieleszko A.J., Chu S.J., Zhang Y. (2014). On the interfacial fracture of porcelain/zirconia and graded zirconia dental structures. Acta Biomater..

[28] Zhang Y., Ma L. (2009). Optimization of ceramic strength using elastic gradients. Acta Mater..

[29] Zhang Y., Chai H., Lawn B.R. (2010). Graded structures for all-ceramic restorations. J. Dent. Res..

[30] Toraya H., Yoshimura M., Somiya S. (1984). Calibration curve for quantitative analysis of the monoclinic-tetragonal ZrO_2_ system by X-ray diffraction. J. Am. Ceram. Soc..

[31] Garvie R.C., Nicholson P.S. (1972). Phase analysis in zirconia systems. J. Am. Ceram. Soc..

[32] Tong H., Tanaka C.B., Kaizer M.R., Zhang Y. (2016). Characterization of three commercial Y-TZP ceramics produced for their high-translucency, high-strength and high-surface area. Ceram. Int..

[33] Catramby M.F., do Vale A.L., Dos Santos H.E.S., Elias C.N. (2021). Effect of sintering process on microstructure, 4-point flexural strength, and grain size of yttria-stabilized tetragonal zirconia polycrystal for use in monolithic dental restorations. J. Prosthet. Dent..

[34] Chai H., Mieleszko A.J., Chu S.J., Zhang Y. (2018). Using glass-graded zirconia to increase delamination growth resistance in porcelain/zirconia dental structures. Dent. Mater..

[35] Yan M., Csík A., Yang C.C., Luo Y., Fodor T., Ding S.J. (2018). Synergistic reinforcement of surface modification on improving the bonding of veneering ceramics to zirconia. Ceram. Int..

[36] Mahadik D.B., Rao A.V., Rao A.P., Wagh P.B., Ingale S.V., Gupta S.C. (2011). Effect of concentration of trimethylchlorosilane (TMCS) and hexamethyldisilazane (HMDZ) silylating agents on surface free energy of silica aerogels. J. Colloid Interface Sci..

[37] Neis C.A., Albuquerque N.L., Albuquerque Ide S., Gomes E.A., Souza-Filho C.B., Feitosa V.P., Spazzin A.O., Bacchi A. (2015). Surface treatments for repair of feldspathic, leucite - and lithium disilicate-reinforced glass ceramics using composite resin. Braz. Dent. J..

[38] Jin C., Wang J., Huang Y., Yu P., Xiong Y., Yu H., Gao S. (2022). Effects of hydrofluoric acid concentration and etching time on the bond strength to ceramic-coated zirconia. J. Adhes. Dent..

[39] Bitencourt S.B., Hatton B.D., Bastos-Bitencourt N.A., Dos Santos D.M., Pesqueira A.A., De Souza G.M. (2022). Silica deposition on zirconia via room-temperature atomic layer deposition (RT-ALD): Effect on bond strength to veneering ceramic. J. Mech. Behav. Biomed. Mater..

